# Credibility Assessment Method of Sensor Data Based on Multi-Source Heterogeneous Information Fusion

**DOI:** 10.3390/s21072542

**Published:** 2021-04-05

**Authors:** Yanling Feng, Jixiong Hu, Rui Duan, Zhuming Chen

**Affiliations:** School of Information and Communication Engineering, University of Electronic Science and Technology of China, Chengdu 611731, China; yanlingfeng@std.uestc.edu.cn (Y.F.); jixionghu@std.uestc.edu.cn (J.H.); duan_rui@uestc.edu.cn (R.D.)

**Keywords:** credibility, sensor, evaluation, multi-source, fusion

## Abstract

The credibility of sensor data is essential for security monitoring. High-credibility data are the precondition for utilizing data and data analysis, but the existing data credibility evaluation methods rarely consider the spatio-temporal relationship between data sources, which usually leads to low accuracy and low flexibility. In order to solve this problem, a new credibility evaluation method is proposed in this article, which includes two factors: the spatio-temporal relationship between data sources and the temporal correlation between time series data. First, the spatio-temporal relationship was used to obtain the credibility of data sources. Then, the combined credibility of data was calculated based on the autoregressive integrated moving average (ARIMA) model and back propagation (BP) neural network. Finally, the comprehensive data reliability for evaluating data quality can be acquired based on the credibility of data sources and combined data credibility. The experimental results show the effectiveness of the proposed method.

## 1. Introduction

Wireless sensor networks (WSN) have become popular in various areas [1,2,3,4]. For example, in agricultural production, WSN are used to monitor the growth environment and status of crops; in terms of industrial safety, WSN are applied to monitor the safety of dangerous working environments such as coal mines, oil drilling, and nuclear power plants to ensure the safety of workers. However, due to the interference of many external factors such as sensor aging, instrument failure, measurement methods, and human interference, the data collected by sensors may not be precise or reliable [5]. The existence of these unreliable data will lead to inefficient data utilization, waste of economic costs, and even serious decision-making errors. Thus, false alarms and missed alarms, which reduce the performance of the monitoring system greatly, will be caused by the unreliable monitoring system. If the credibility of the original data can be evaluated effectively, then the unreliable and low-quality data can be detected from the original data, contributing to the data processing, and the remaining high-reliability data will improve the early warning capabilities of the supervision and inspection system, and thereby the safety of people’s life and property can be guaranteed to the maximum extent. For real event data, if the data collected by data sources reflect the real environment, this method considers the data as credible, which could be used in further data analysis. When the data of multiple sensors are interfered, this method will not be able to make an effective evaluation of the data, so the event data and unreliable data cannot be handled.

In order to evaluate the credibility of data, scholars have conducted many methodological studies. For example, Marsh [6] first proposed a mathematical model for calculating credibility, and at the same time, explained the definition of data credibility. He considered that credibility is a way to understand a complex environment, a means to provide additional robustness to independent subjects, and an effective indicator to make judgments based on the experience of others.

According to the above definition, Marsh suggested several important directions for subsequent research. Galland [7] presented three fixed-point algorithms based on the complexity of the data source to evaluate the credibility of the data provided by the data source. Furthermore, based on the experimental results, he designed two schemes to enhance robustness of the method: establishing weights for different data sources and using prior knowledge. Aiming at the uncertainty and conflict of multi-source data, Guo [8] et al. used a reliability evaluation framework based on evidence theory, which combines prior information and context information, and the algorithm can be improved by correlating with more information. Jikui Wang et al. [9] suggested an evaluation method of data with Bayesian estimation theory, which relies too much on the accuracy of the prior data of the data source. Scholars such as Fernando [10] aimed at the problem of evaluating the credibility of sensors for autonomous driving systems by machine learning to build a framework for data credibility, and using Lidar to verify the system. However, models are directly established from the data in the above algorithms, without constructing the spatio-temporal relationship between the data sources. Thus, it is not possible to exploit the advantage that the correlation between data sources can handle the uncertainty of the data. For the literature [11], Xinjian Wan et al. designed a credibility measurement model, which considered the relationship between data sources, in light of the problem that the credibility of air quality monitoring data cannot be evaluated. Hence, this was our main comparison method in this article.

Multi-source data fusion is a processing means for multi-source data to derive estimates and judgments from the original data sources through knowledge-based reasoning and recognition to enhance the confidence of data, improve reliability, and decreased uncertainty, with the advantages of comprehensive information, high fault tolerance, reduced cost, and broader coverage. It is a complex estimation process. In order to make full use of the information in the image fusion process, Xue J et al. [12] developed a data fusion method based on Bayesian, and finally the fused image was obtained by maximum a posteriori probability (MAP). However, the Bayesian criterion is greatly limited in engineering applications due to the need to obtain the prior probability of each data source, and this requirement is difficult to know in advance. In [13], three multi-sensor data fusion algorithms based on Kalman filter, namely state vector fusion (SVF), measurement fusion (MF), and gain fusion (GF) are implemented in a tracking system. Results showed that the Kalman filter-based state vector fusion algorithm performed well comparatively for the system. Although the Kalman filter has good performance in target tracking applications, it also has the problem of poor real-time performance. L.Z. et al. [14] presented weather recognition methods based on the weighted fusion of weather-specific features for classifying five types of weather. It is simple to achieve the weighted average method, but the real-time performance is also poor. Moreover, some algorithms do not take the fluctuation of data collected by sensors in the time series into account, and do not reflect the accuracy of data fusion. The BP neural network has the advantages of simple design, fast training speed, strong self-adaptive ability, and it has good fault tolerance because it does not cause a great impact on the global training results after its local or partial neurons are damaged [15]. Here, this paper proposes a new comprehensive credibility of data measurement (CDM) method, which combines the correlation between data sources and the fusion algorithm. This method makes full use of the relationship between data sources to further enhance the robustness. It also utilizes the historical data of the target data source and the multi-source heterogeneous data to improve the accuracy of the evaluation. Based on the above characteristics of the BP neural network, the fusion algorithm was selected as a BP neural network. Moreover, the ARIMA model [16] has the characteristics of stable prediction, simple, and easy implementation. Therefore, the model based on ARIMA and BP neural network can provide stable, real-time, and accurate assessment of the credibility of data.

The rest of this article is organized as follows. The definition of credibility and the components of data credibility are introduced in Section 2, and the basic concepts of the ARIMA model and BP neural network are described in detail. The proposed method through examples is verified and the experimental results are presented in Section 3. Our conclusion and next work plan are presented in Section 4.

## 2. Materials and Methods

Currently, there is no clear and accepted definition of the credibility of data. Pinyol I [17] discussed the objective aspect of credibility and argued that credibility can be regarded as a decision in the process of interacting with another party. Sabater J [18] analyzed the user’s credibility evaluation mechanism of eBay, Amazon Auctions, and OnSale Exchange, and noted that the credibility models of these online markets all used single data that do not depend on contextual information. In [19], the authors considered that data credibility is determined by a combination of data integrity, consistency, and data accuracy. Consistency indicates whether the logical relationships between related data are correct, data integrity describes the severity of data loss in the dataset, and accuracy demonstrates how close the measured data are to the real data. For this article, the credibility of the data provided by the data source was considered to be the degree to which the measured object in the expected environment reflects the true state. That is, the discussion focused on the accuracy index as an important index to measure the performance of the data credibility measurement model.

First, there is an overview of the methodology. Furthermore, there is an introduction of the symbols, a definition of comprehensive data credibility, a meaning, and the metrics of the parameters in formulas are described in detail. Finally, we briefly review the related algorithms presented.

### 2.1. Overview

**Figure 1:**
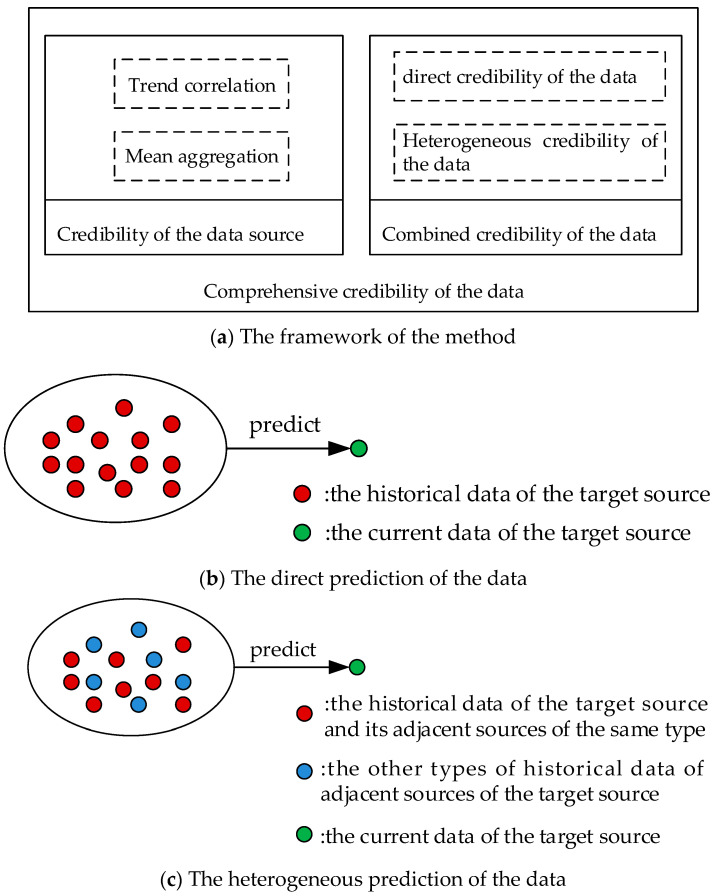
Overall flow chart. (**a**) The framework of the method. (**b**) The direct prediction of the data. (**c**) The heterogeneous prediction of the data.

In order to clarify the relationship of the different factors, we present the framework of the method, and the specific process of the method is shown in Figure 1.

Figure 1a shows the framework of the method, which illustrates the relationship of the credibility of the data source and the combined credibility of the data, and shows the components of the different type of credibility. Figure 1b demonstrates the process of predicting data directly through the time correlation of data. Figure 1c shows the process of predicting the heterogeneous data through the spatio-temporal correlation.

### 2.2. Symbols

In this section, we describe the symbols used in this paper. The symbols and descriptions are shown in Table 1.

### 2.3. Definition of Comprehensive Data Credibility

The evaluation method of comprehensive data credibility combines multiple sensors and multiple types of sensors in both temporal and spatial dimensions. Comprehensive data credibility is composed of data source credibility and combined data credibility. The calculation of data credibility is as follows:

(1) $$R_{t}^{i}=\left\{ \begin{array}{ll} S_{t}^{i}\times D_{t}^{i}, & S_{t}^{i}>\Theta \\ 0 & else \end{array}\right.$$

where $R_{t}^{i}$ is the comprehensive data credibility of the data source i; $S_{t}^{i}$ represents the credibility of the data source i; $D_{t}^{i}$ is the combined data credibility of the data source i; and Θ is the credibility threshold of the data source. When the data source is credible, the comprehensive data credibility is the result of multiplying the credibility of the data source and the combined credibility of the data. If the credibility of the data exceeds 0.5, the data is credible, otherwise the data is not credible. In this paper, the data credibility is represented by the continuous value between the interval [0,1]. Next, the credibility of the data source and the combined credibility of the data are elaborated.

### 2.4. Definition of the Credibility of the Data Source

Data source credibility will be calculated through trend correlation and mean aggregation, used to describe whether the data source is credible and whether it is working properly. If the data source is not credible, the credibility of the data is determined to be zero. The formula for the credibility of the data source is as follows.

(2) $$S_{t}^{i}=K_{11}C_{\mathrm{tr}}^{i}+K_{12}C_{ave}^{i}$$

where $S_{t}^{i}$ is the credibility of the data source; $C_{tr}^{i}$ is the trend correlation of the data source i; $C_{ave}^{i}$ is the mean aggregation of the data source i; and $K_{11}$ and $K_{12}$ weighting factors of trend correlation and mean aggregation, respectively, where $K_{11}+K_{12}=1$. The weights are determined based on the importance of trend correlation and mean aggregation. Through multiple iterations, the weights are determined with the highest detection probability and the lowest false alarm rate. The definition of the detection probability and false alarm rate is introduced in the experimental section. The following parameters were the same.

(1) Calculation of trend correlation

Setting a distance threshold φ, this is a Euclidean distance. When the distance between the target data source and a data source is less than the threshold, the data source is regarded as an adjacent data source. Trend correlation refers to the average degree of correlation between the historical data trend of the target data source and its adjacent data sources. If the data source is damaged or does not work correctly, $C_{\mathrm{tr}}^{i}$ will be smaller. The mathematical expression of the trend correlation is as follows.

(3) $$C_{\mathrm{tr}}^{i}=\frac{1}{N}\sum_{j=1}^{N}\left|r(Tr^{i}(d_{s}),Tr^{j}(d_{s}))\right|$$

(4) $$r(Tr^{i}(d_{s}),Tr^{j}(d_{s}))=\frac{\sum_{s=1}^{S}\left(Tr^{i}(d_{s})-\bar{Tr^{i}(d)})(Tr^{j}(d_{s})-\bar{Tr^{j}(d)}\right)}{\sqrt{{\sum_{s=1}^{S}\left(Tr^{i}(d_{s})-\bar{Tr^{i}(d)}\right)}^{2}}\sqrt{{\sum_{s=1}^{S}\left(Tr^{j}(d_{s})-\bar{Tr^{j}(d)}\right)}^{2}}}$$

where $Tr^{i}(d_{s})$ is the trend of a piece of the historical data; r is a correlation function; the data source j is adjacent to the data source i; and N is the number of adjacent data sources of the target data source; the vertical line indicates the absolute value; S is the number of historical data; and $\bar{Tr^{}(d)}$ represents the mean value of the historical data.

(2) Calculation of mean aggregation

Mean aggregation is the degree of aggregation between the average value of the target data source and its neighboring data sources of the same type over the same time. Similarly, it also describes the degree of dispersion of average data between data sources with spatial correlation. For example, when a data source is manually interfered, it is possible that the data trend of the target source is still consistent with that of the neighboring data sources, but the value will decrease, and the mean value will deviate from the mean value of other neighboring data sources, then $C_{ave}^{i}$ will be reduced. The calculation formula for the mean aggregation is as follows:

(5) $$\begin{array}{l} C_{\mathrm{ave}}^{i}=\frac{1}{\left|\frac{1}{M}\sum_{p=1}^{M}\left|Da_{i}-Da_{p}\right|*{\bar{d}}_{ip}-\frac{1}{MN}\sum_{p=1}^{M}\sum_{q=1}^{N}\left|Da_{p}-Da_{q}\right|*{\bar{d}}_{pq}\right|+1} \end{array}$$

(6) $${\bar{d}}_{ip}=\;1-\frac{d_{ip}}{\sum_{p=1}^{M}d_{ip}}$$

where $Da_{i}$ is the average value of the historical data; the data source p is the adjacent sensor of the data source i; M is the number of adjacent sensors of the data source i; the data source q is adjacent to the data source p; and N is the number of adjacent sensors of the data source p; and $\bar{d}$ is the normalized distance factor between data sources.

### 2.5. Definition of the Combined Credibility of Data

Combined credibility of the data consists of direct credibility of the data and heterogeneous credibility of the data. The formula for the combined credibility of the data is as follows:

(7) $$D_{t}^{i}=(K_{21}F_{t}^{i}+K_{22}M_{t}^{i})$$

where $D_{t}^{i}$ is the combined credibility of the data provided by data source i; $F_{t}^{i}$ is the direct credibility of the data provided by data source i; $M_{t}^{i}$ is the Heterogeneous credibility of data; and $K_{21}$ and $K_{22}$ are the weighting factors of direct credibility of the data and heterogeneous credibility of the data, respectively, where $K_{21}+K_{22}=1$.

(1) Calculation of direct credibility of the data

Direct credibility of the data is the degree of membership of the measured value to the theoretical real value. Namely, the historical data of the data source is used for model fitting based on the ARIMA model, and then the theoretical real value at the current moment is predicted according to the time correlation based on the fitting function. The directly credibility is obtained based on the measured value and theoretical real value. The formula for the direct reliability of the data is as follows.

(8) $$F_{t}^{i}=\{\begin{array}{cc} 1 & \Delta f_{t}^{i}\leq \eta \;\\ \frac{3\eta -\Delta f_{t}^{i}}{2\eta } & \eta <\Delta f_{t}^{i}<3\eta \\ 0 & \Delta f_{t}^{i}>3\eta \end{array}$$

(9) $$\Delta f_{t}^{i}=|Rp_{t}^{i}-Sp_{t}^{i}|$$

where $Rp_{t}^{i}$ is the measure data of the data source i; $Sp_{t}^{i}$ is the theoretical real value (direct prediction data) based on the historical data; and η  is the credibility threshold of the data.

(2) Calculation of heterogeneous credibility of the data

Heterogeneous credibility of the data is the degree of membership of the measured value to the theoretical real value. Multi-source heterogeneous data, which are from the target data source and its multiple neighboring heterogeneous data sources within the same period of time, are used in data prediction currently, based on the BP neural network. Then, heterogeneous reliability is calculated according to theoretical real value and measured value. The formula for the heterogeneous reliability of the data is as follows.

(10) $$M_{t}^{i}=\{\begin{array}{cc} 1 & \Delta m_{t}^{i}\leq \eta \;\\ \frac{3\eta -\Delta m_{t}^{i}}{2\eta } & \eta <\Delta m_{t}^{i}<3\eta \\ 0 & \Delta m_{t}^{i}>3\eta \end{array}$$

(11) $$\Delta m_{t}^{i}=|Rp_{t}^{i}-Mp_{t}^{i}|$$

where $Mp_{t}^{i}$ is the theoretical real value (heterogeneous prediction data) of data source i at time t by fusing the data of the adjacent data sources.

#### 2.5.1. Autoregressive Integrated Moving Average Model

- Overview

In this part, we introduce the proposed time series forecasting model. The model can extract the historical data trend of the data source to predict the future data. First of all, the parameters used in this section are explained. The parameters and descriptions are shown in Table 2.

**Figure 2:**
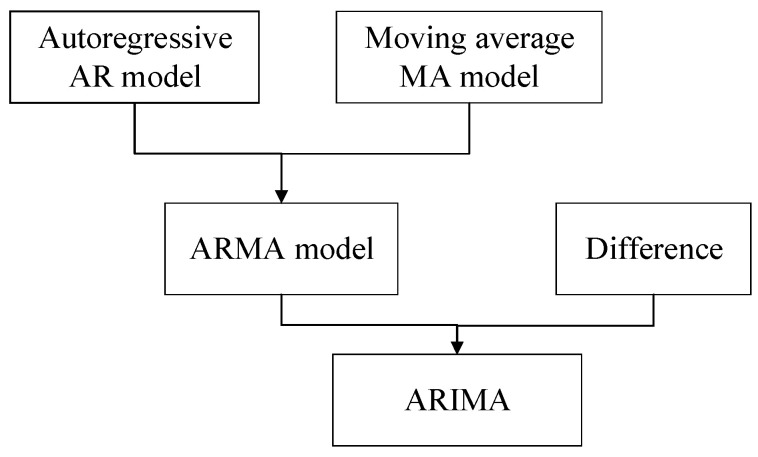
The relationship between ARMA and ARIMA.

The full name of the ARIMA is called the autoregressive integrated moving average model, which is also recorded as ARIMA(p,d,q). ARIMA is the most common statistical model used for time series forecasting [20,21], which is an extension of the ARMA(p,q) model [22,23,24]. The relationship between the two models is shown in Figure 2.

The ARMA model consists of an autoregressive model and a moving average model, where, p and q are the two parameters of the ARMA model, and the meanings of these parameters are shown in Table 2. The mathematical representation of the ARMA model is as follows:

(12) $$y_{t}=\mu +\sum_{i=1}^{p}\varphi_{i}y_{t-i}+e_{t}+\sum_{j=1}^{q}\theta_{j}e_{t-j}$$

where $y_{t}$ is the prediction value at time t; μ is a constant; φ is the coefficient of the AR model; θ is the coefficient of the MA model; and $e_{}$ is the error between the predicted value and the measured value.

Compared with the ARMA model, the ARIMA model adds a difference algorithm, the purpose of which is to convert a non-stationary sequence into a stationary sequence to meet the stationary demand of ARIMA. Among them, p, d, q, are the three parameters of the ARIMA model. The meaning of each parameter is shown in Table 2. The mathematical representation of the ARIMA model is as follows:

(13) $$y_{t}^{\left(d\right)}=\mu +\sum_{i=1}^{p}\varphi_{i}y_{t-i}^{\left(d\right)}+e_{t}+\sum_{j=1}^{q}\theta_{j}e_{t-j}$$

where $y_{t}^{\left(d\right)}$ is the predicted value of the d-order difference of the data at time t.

**Figure 3:**
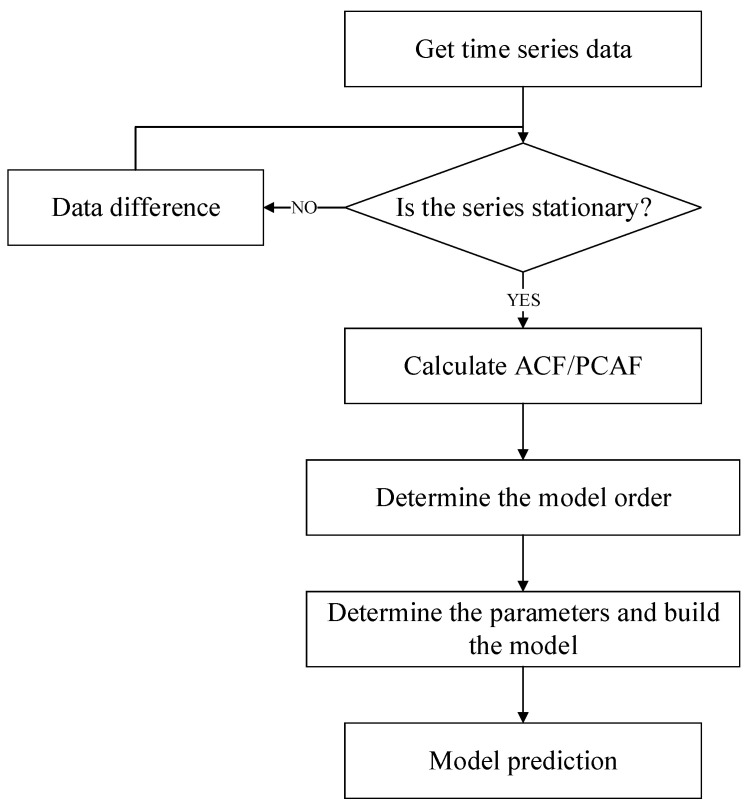
Program flow diagram of the model.

It is important to establish an appropriate model for the accuracy of data prediction. There are three steps to build a time series prediction model, namely, model ordering. The model flow chart is shown in Figure 3.

1. First, it is necessary to judge whether the time series data are stable, and if they are not stable, corresponding processing is required such as difference and logarithmic operations;
2. Second, if the data series is stable, the next step is to determine the order p, q, which can be determined artificially by calculating the autocorrelation function (ACF) and the partial autocorrelation function (PACF);
3. Third, since there is a certain subjective component in the process of determining the order in the previous step, it is necessary to verify whether the parameters are reasonable through the index function, and the index function is selected as the Akaike Information Criterion (AIC)/Bayesian Information Criterion (BIC); and
4. Finally, after the model is established, the future data can be predicted through the model.

- Parameter Selection

**Figure 4:**
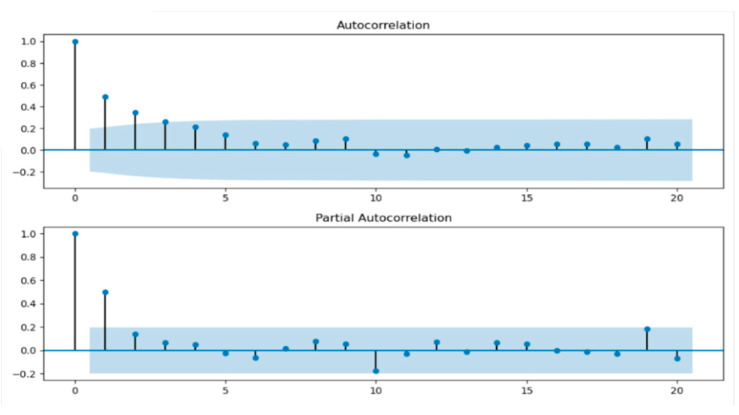
The visualization of ACF and PACF.

**Figure 5:**
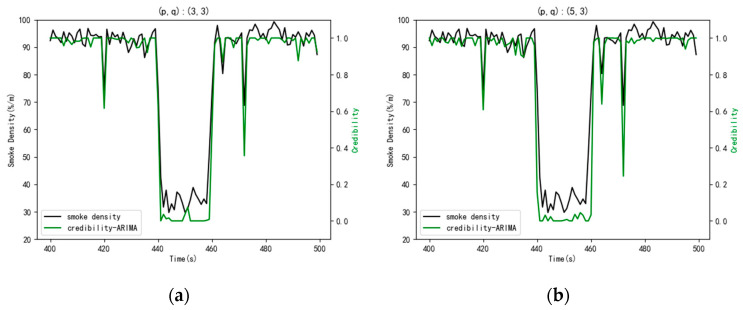
The comparison of the ARIMA model with two different parameters. (**a**) The parameters of (p,q) were (3,3); (**b**) the parameters of (p,q) were (5,3).

In the ARIMA model, the parameters of (p,q) can be determined artificially through the ACF and PACF, and the visualizations are shown in Figure 4. Finally, (p,q) were determined as (3,3). Due to the strong subjective factors in order determination through ACF and PACF, we can again determine the order according to the information criterion function method. In this experiment, the AIC index function was selected, and the parameters (p,q) were selected as (5,3). Then, the results of the data credibility were obtained by the model with two different parameters and the results are shown in Figure 5.

Observing Figure 5a,b, the difference between the results was not very great. Both sets of parameters can be used. In this paper, the parameters of (p,q) are finally confirmed as (5,3).

#### 2.5.2. Back Propagation (BP) Neural Network

- Overview

**Figure 6:**
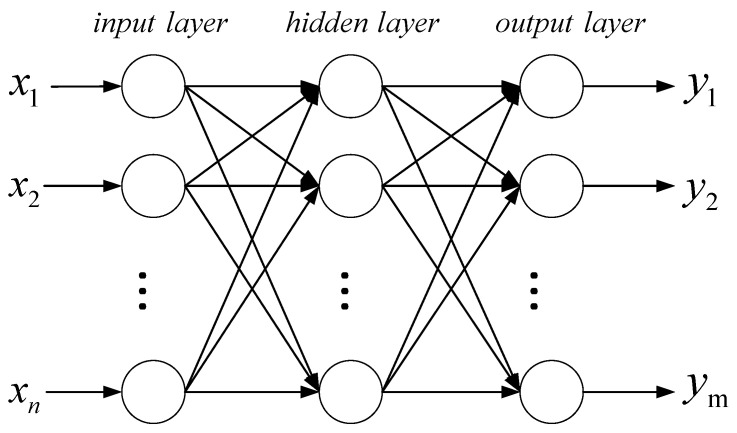
Program flow diagram of the model.

The BP neural network is a multi-layer feedforward neural network [25,26,27] including an input layer, an output layer, and one or more hidden layers. The main characteristics are signal forward propagation and error back propagation. Specifically, the training process of the neural network can be divided into two stages. The first stage is signal forward propagation, where the input of the network is from the input layer through the hidden layer before finally obtaining the result from the output layer; in the second stage, error back propagation, error and gradient go from the output layer to the hidden layer and finally back to the input floor. The basic simple structure is shown in Figure 6.

Suppose this network has n inputs, m outputs, and a hidden layer, among which there are s neurons in the hidden layer. Suppose the output of the hidden layer is $a_{j}$, the bias is $\theta_{j}$, the weight is $w_{ij}$, and the activation function is $f_{1}$. The output of the output layer is $y_{k}$, the bias is $\theta_{j}$, the weight is $w_{jk}$, and the activation function is $f_{2}$. The mathematical expression for calculating the output of the hidden layer is as follows:

(14) $$a_{j}^{}=f_{1}\left(\sum_{i=1}^{n}w_{ij}x_{i}+\theta_{j}\right)(j=1,2,\ldots ,s)$$

The mathematical expression for calculating the output of the output layer is as follows:

(15) $$y_{k}=f_{2}\left(\sum_{j=1}^{s}w_{jk}a_{j}+\theta_{k}\right)(k=1,2,\ldots ,m)$$

The error function of the network is defined as follows:

(16) $$e=\sum_{k=1}^{m}{\left(y_{k}-t_{k}\right)}^{2}$$

where $t_{k}$ is the true value (expected value) of the output.

The objective of the training process is to reduce the error as much as possible. The forward process is to calculate $y_{k}$ and the error e, and then back-propagate the error to modify the weight coefficient of each layer, repeat iteratively, and until the training is ended

- Parameters Selection

**Figure 7:**
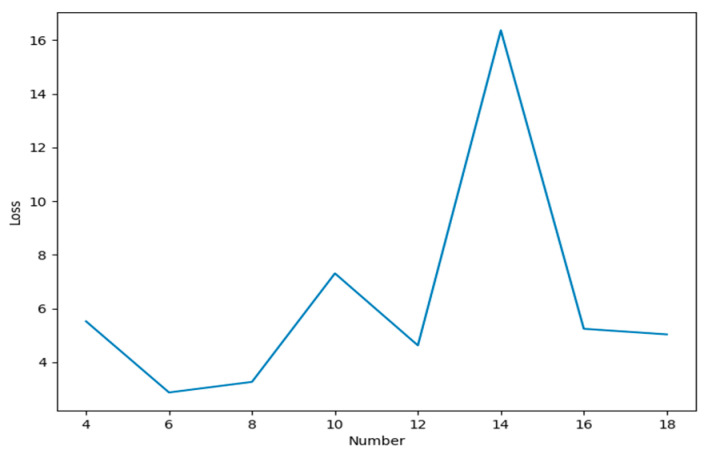
The influence of the different number of neurons on the result.

This neural network also takes advantage of the spatio-temporal relationship be-tween data sources, and uses data from neighboring data sources as input for learning. During the learning process, the number of neurons in each layer has a greater impact on the training results as it not only affects the training speed, but also affects the training accuracy. Here, on the basis of other parameters unchanged, we set the number of hidden layers of the network to 2, and the influence of the number of different neurons in each layer on the training accuracy is shown in Figure 7.

In Figure 7, it can be seen that when the number of neurons in each hidden layer is six, the error was the smallest. Therefore, the number of neurons was six, and the other parameters were set as follows: Learning rate *ꞵ* = 0.01, batchsize=12, and epoch=20.

## 3. Results and Discussion

A simulation was conducted to verify the effectiveness of the proposed method, and whether the credibility measurement method has the ability to detect unreliable data from the original dataset.

### 3.1. Experiment Design

**Figure 8:**
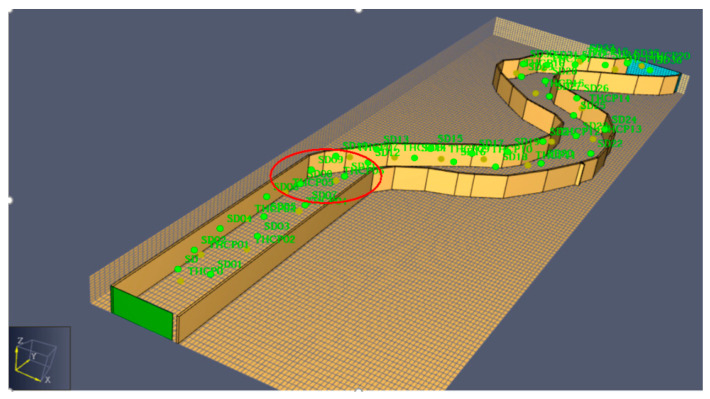
Software model diagram.

The selected data of the simulation experiment were from the alleyway smoke simulation dataset generated by Pyrosim software, which was developed abroad for fire simulation. The distribution of the dataset is shown in Figure 8. In the process of data collection, two kinds of data, smoke density and temperature, were collected respectively.

**Figure 9:**
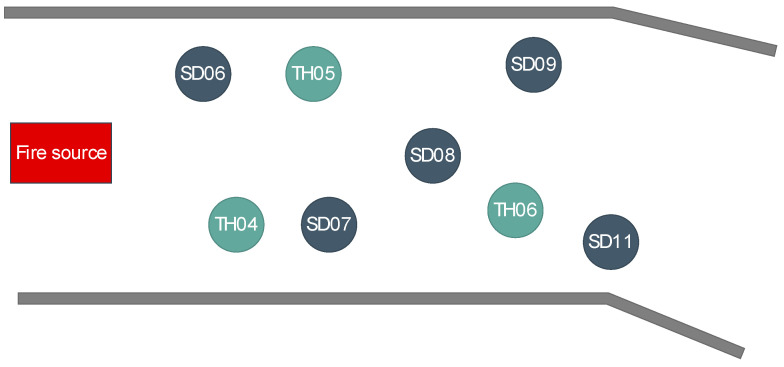
Top view of distribution.

The red circle area is the distribution of the data sources used in the experiment. In order to see the correlation distribution of the data sources more clearly, the top view of the region is shown in Figure 9.

The dataset contained 1000 sets of data provided by eight data sources. Table 3 shows the data samples.

Many parameters are mentioned in this paper, which include the weight of trend correlation, the weight of mean aggregation, the weight of data direct credibility, the weight of data heterogeneous credibility, the credibility threshold of the data, and the credibility threshold of the data source. In this experiment, these parameters were set based on subjective judgment and experience, and the parameters were specifically set as shown in Table 4.

Before the experiment, the data of smoke density and temperature were considered to be completely reliable after manual screening and verification. Based on this data, the artificial interference to the data was divided into normal interference and large abnormal interference, and the purpose of the experiment was to judge whether the presented method could identify abnormal interference and give the corresponding results of data credibility.

In this dataset, 100 sets of data in the time of T499–T500 were selected as the data to be evaluated in data samples, in which SD08 was taken as the target data source, and SD08 was interfered with between T499 and T500, according to the previously designed data interference type. Other data sources were all neighboring data sources of the target data source, where the neighboring data sources with the same type as the target data source are called neighboring data sources of the same type.

**Figure 10:**
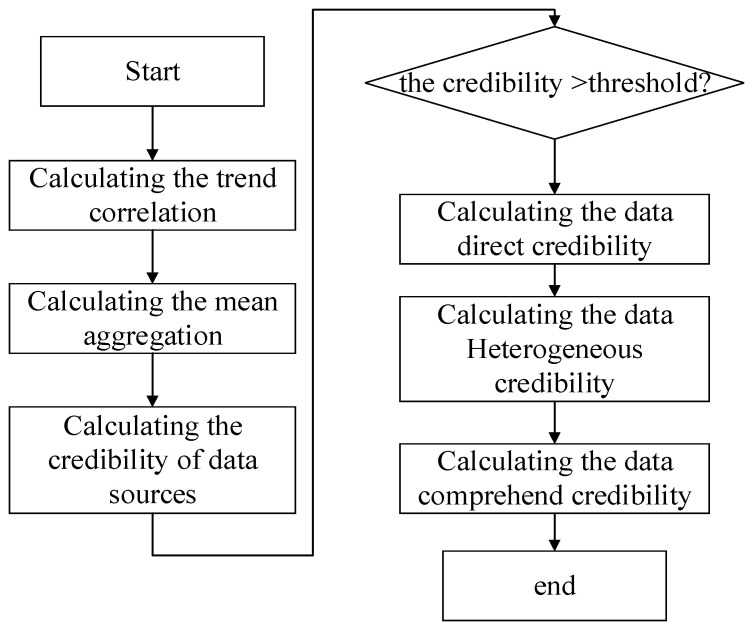
The flow chart of the experimental process.

The flow chart of the entire experimental process is shown in Figure 10. First, the trend correlation of the data was calculated according to Equation (3). Before the calculation, the historical data interval needs to be selected. Through tests, when the data interval length was 10, it could best reflect the real situation of the environment in real time without reducing the accuracy. Moreover, Equation (5) was used to calculate the mean aggregation of the data. Based on the trend correlation and mean aggregation, the credibility of the data source was calculated by Equation (2). Second, using the ARIMA models, the direct credibility of data was acquired by Equations (8) and (9) using the BP neural network, and the heterogeneous data reliability was obtained according to Equations (10) and (11). Furthermore, based on the direct credibility of data and heterogeneous credibility of the data, the data combined credibility was calculated by Equation (7). Finally, the comprehensive data credibility was achieved according to Equation (1).

**Figure 11:**
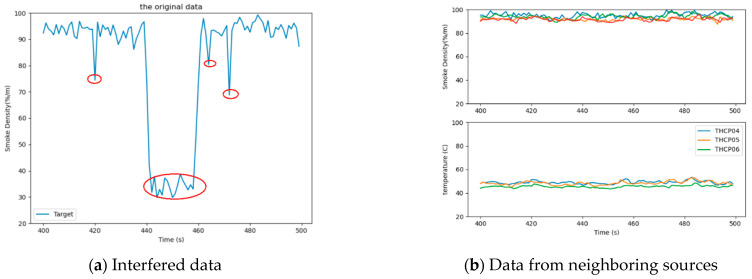
Data visualization. (**a**) Interfered data; (**b**) Data from neighboring sources.

Next, the interfered target data and the data generated by its neighboring data sources are demonstrated in Figure 11.

The data in red circles in Figure 11a are the artificially disturbed data. The remaining data are normal data. Figure 11b is the data from the adjacent sources.

### 3.2. Analysis of Results

#### 3.2.1. Experiment Result

**Figure 12:**
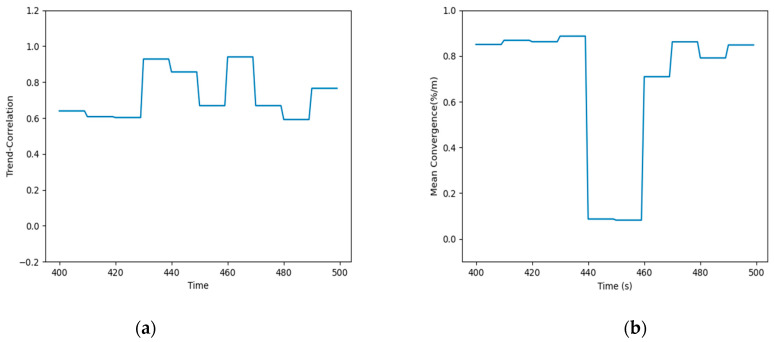
The credibility of the data source. (**a**) The trend correlation; (**b**) The mean aggregation.

In order to evaluate the performance of the algorithm, we defined the detection probability and false alarm rate of incredible data, which can be seen in Equations (17) and (18). Figure 12 shows the data trend correlation and mean aggregation. In Figure 12a, the trend correlation was mainly maintained between [0.6, 0.8], which showed a downward trend, but did not reach a low value during T440–T460. This is because the interference for sensors only makes the value smaller, but the fluctuation of the value is not completely changed. Hence, the trend correlation during this period was not too low. However, for the mean aggregation, the target data were at a lower level for a longer time than the data from the neighboring sources of the same type between time T440 and T460. The difference between the data became larger, leading to a smaller mean aggregation. Figure 12b, indeed, shows the corresponding change.

(17) $$p_{d}=\frac{d}{D}$$

(18) $$p_{fa}=\frac{f}{F}$$

In the above equations, $p_{d}$ is the detection probability; d is the number of incredible data detected correctly; D is the total number of incredible data added; $p_{fa}$ is the false alarm rate; and f is the number of incredible data detected incorrectly. F is the total number of credible data.

**Figure 13:**
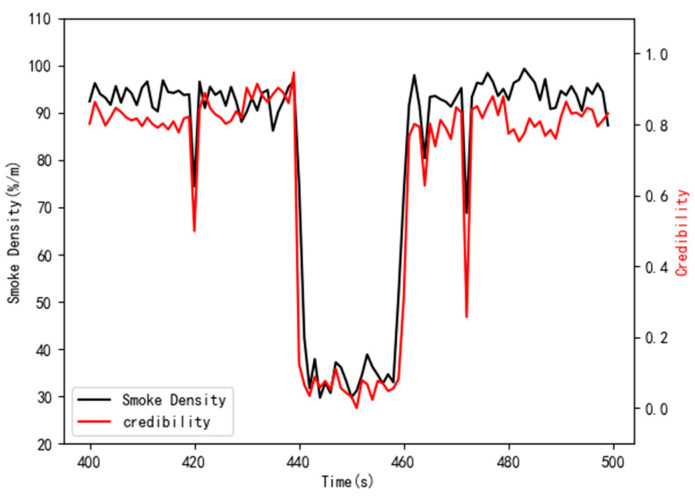
Assessment results of comprehensive data credibility.

After determining the parameters of each algorithm, the parameters were also configured according to Table 4, and then the data credibility measurement model based on multi-source heterogeneous information fusion was built. During the experiment, three sets of single-point error data and 21 sets of error data in the time T440–T460 were artificially constructed to simulate the situation where the data source provided the error data, mainly to prove the model’s ability of detecting unreliable data. The evaluation results are presented in Figure 13.

In Figure 13, the credibility of the data remained stable between time T400 and T420. As the target data were not disturbed in this time, the data are credible. At the time of T420, T464, and T472, the credibility of the data dropped significantly, especially at the time of T420 and T472. Due to the great interference, the data credibility dropped below 0.5. During the time period T440–T460, the target data source was not credible, and the data credibility directly dropped to zero because the data were changed too large. The experimental results showed that when the original data were modified manually, the model could detect the wrong data and could dynamically adjust the credibility of the data in a larger and smaller range. When the measured data differs greatly from the real data, the credibility of the data will drop below 0.5 or even zero.

#### 3.2.2. Performance Comparison

**Figure 14:**
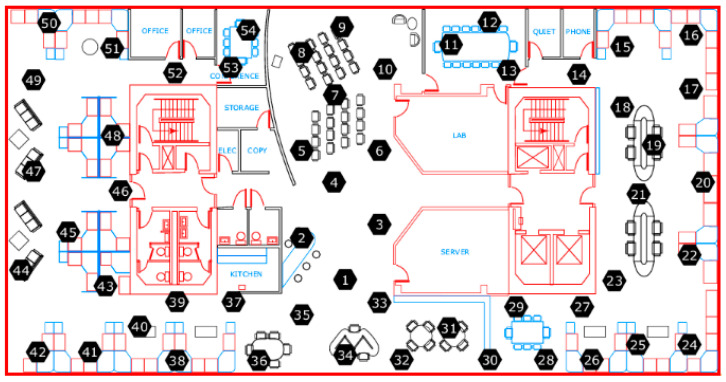
Location distribution of the sensor nodes.

In order to verify the advantage of the spatio-temporal relationship in the BP neural network for predicting data, some neural network prediction models were used to compare the performance in the simulations. These models are explained below. This comparison used two datasets, one was the dataset generated by Pyrosim software, and the other was the Intel indoor dataset [28], as a benchmark dataset to study the data prediction in the wireless sensor networks. This Intel indoor dataset consisted of 54 sensor nodes distributed in the laboratory, and the location distribution of 54 sensor nodes is shown in Figure 14. The Intel Berkeley Research lab used the Mica2Dot wireless sensor as the collection node to collect the four physical quantities of ambient temperature, humidity, light, and battery voltage.

Here, we used node 13 as an example and as the center, and then selected node 11, node 12, and node 14 as its neighboring sources. Considering the data correlations of signal-node and multi-node, which are shown in Table 5 and Table 6, the paper selected the temperature and humidity data of node 13, the temperature data of node 11, node 12, and node 14, and the humidity data of node 12 and node 14 as the input data of the BP neural network, which were used to predict the temperature data of node 13.

**Figure 15:**
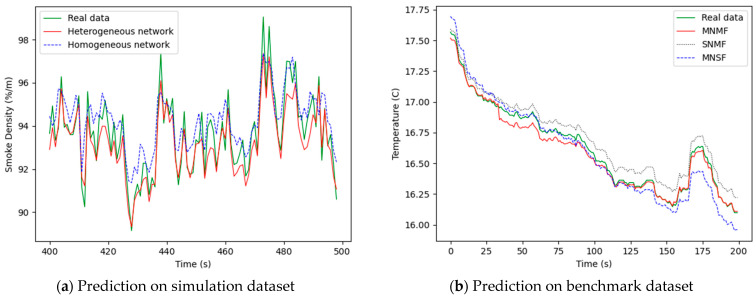
Prediction on two datasets. (**a**) Prediction on the simulation dataset. (**b**) Prediction on the benchmark dataset.

For the simulation dataset, according to the different input data, we can divide the network into the homogeneous network and heterogeneous network. The BP neural network is equivalent to thee heterogeneous network because of using different types of data, smoke data, and temperature data of different sources. Figure 15a shows the data prediction of the different network. For the benchmark dataset, the BP network in this paper selected the multi-data of multi-node, so it is called the multi-node multi-feature (MNMF) model here. In contrast to this model, a signal-node multi-feature (SNMF) model and a multi-node single-feature (MNSF) are presented. The SNMF model uses the data of a signal node, for example, the temperature and humidity data of node 13. The MNSF model uses the same type of data of a multi-node, for example, the temperature data of node 13, node 11, node 12, and node 14. Figure 15b shows the data prediction of the three prediction models. The mean-square error (MSE) is used as evaluation indicator to evaluate the prediction model. The formula for evaluation standard is shown as Equation (19).

(19) $$MSE(y,y^{*})=\frac{1}{m}\sum_{i=1}^{m}{\left(y_{i}-y_{i}^{*}\right)}^{2}$$

In the above equation, $y_{i}$ is the true value; $y_{i}^{*}$ is the predicted value; and m is the number of samples. The calculated evaluation indicators are shown in Table 7.

Figure 15a shows that the heterogeneous network had a better performance than the homogenous network, and Figure 15b shows that the MNMF model had better prediction than the other models. It can be seen from Figure 8 that the heterogeneous network and the MNMF model both had a low prediction error and a high prediction accuracy. Moreover, the heterogeneous network and the MNMF model used the spatio-temporal relationship between different data sources and fused heterogeneous information. This experiment proved that the BP neural network in this paper has a great performance for predicting data.

**Figure 16:**
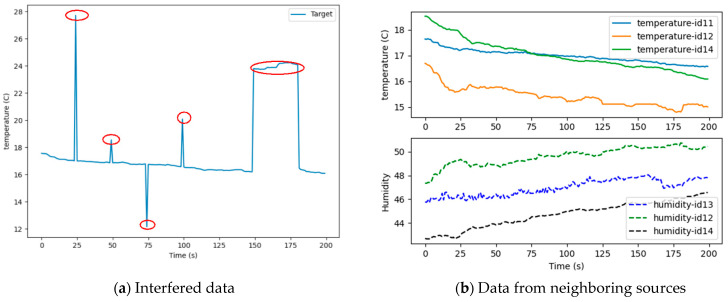
Data visualization. (**a**) Interfered data. (**b**) Data from neighboring sources.

**Figure 17:**
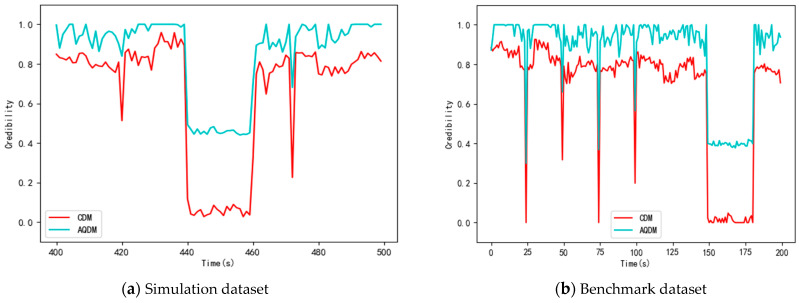
Comparison of credibility measurement models. (**a**) Simulation dataset. (**b**) Benchmark dataset.

According to the analysis in Section 3.2.1, the feasibility and validity of the proposed model were verified. In this section, the methodology in this paper is compared with the Air Quality Credible Measurement Model (AQCM) in [11]. In order to further verify the effectiveness of the method, we again selected the Intel Lab data as the benchmark to study. The interfered target data and the data generated by its neighboring data sources of the benchmark dataset are demonstrated in Figure 16. The comparison result based on the two datasets is depicted in Figure 17.

In Figure 17a, the vertical axis represents the data credibility, and the horizontal axis expresses the time period. At T420, T464, and T472, the CDM model reflected the current state of the data more accurately than the AQCM model. For the AQCM model, only at T472 did the credibility of the data decrease significantly, but it was not less than 0.5. Therefore, the data at time T420, T464, and T472 were judged as credible, which is not consistent with the real situation. In the T440–T460 time period, the evaluation results of the two methods were less than 0.5, and the data were considered to be unreliable, whereas the evaluation result of the methodology in this paper was closer to zero. Figure 17b shows that the CDM model had a better evaluation performance than the AQDM model, especially at times of T50 and T100. For the temperature of node 13 in the benchmark dataset, the data at time T50 and T100 were unreliable. However, the AQDM model made the data reliable. In time period of T150–T180, the evaluation result of the AQDM was closer to zero. It can be seen from Figure 16 that the CDM model had an accurate evaluation and a flexible capability of assessment.

#### 3.2.3. Extent Experiment

**Figure 18:**
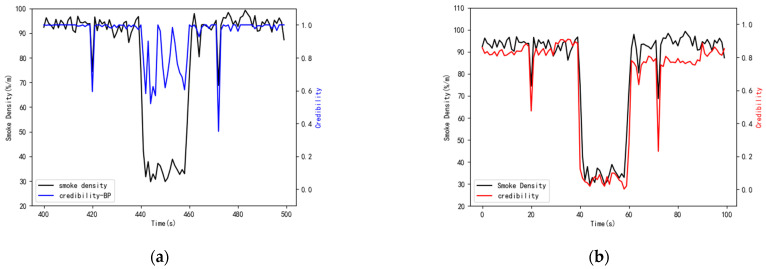
The evaluation result of data credibility. (**a**) Results of the BP assessment of data credibility. (**b**) Results of the comprehensive data credibility.

The purpose of this extended experiment was to verify the robustness of the model. The data source SD11 adjacent to the target source was also interfered in the same time period of T440–T446. The CDM model was applied to this situation, and the results are demonstrated in Figure 18.

Figure 18a shows the credibility of heterogeneous data when two data sources are interfered at the same time. During T440–T460, as the input data of the BP neural network suffer from the same interference, this will cause the network output to also be disturbed, therefore, the evaluation results of data credibility will also be disturbed. In Figure 18b, the comprehensive evaluation of data credibility in T440–T460 were not destroyed, precisely because the correlation between data sources was utilized. The mean aggregation between the target source and other adjacent sources was still close to 0, so that the data credibility in T440–T460 approached 0, which verified that the model also had good robustness.

## 4. Discussion

This paper used two kinds of data credibility—a direct and a heterogeneous credibility. The advantages relative to the individual use of one of them are shown below. The direct credibility utilized the time relationship through the historical data of the target source. Compared with the historical data of other sources, historical data and current data of the same target source had the greatest correlation in normal condition. Therefore, it is necessary to assess the credibility of the data in normal condition. Heterogeneous credibility utilizes the spatio-temporal relationship of the data sources, adding different dimensions to improve the evaluation accuracy. This is vital when the historical data of the target source is not reliable, if the source has been damaged. The combination of the direct and the heterogeneous credibility makes the method more accurate and more robust than the individual use of one of them.

Multi-sensor data fusion is an efficient tool for improving the performance because of the spatio-temporal relationship between data sources. However, there are some challenges and future work to be discussed.

(1) Improvements of the application of the method

In this work, we proposed a method that could effectively utilize the spatio-temporal relationship of the data sources. In order to simplify the problem, we assumed that data integrity and time lag between data were not considered in our experimental set. However, in field measurement, missing data is one of the most common problems. When sources lose data, the performance of this method will be harmed. Due to the hugeness and redundancy of wireless sensor network data, we should apply proper data preprocessing methods to deal with data missing and other data problems. In general, data interpolation is an effective method including quadratic interpolation, cubic interpolation, linear interpolation, and so on.

(2) Development of the fusion algorithm

Each fusion method has its own set of advantages and limitations. The combination of several different fusion schemes has been approved as a useful strategy that may achieve a better performance of results [29]. However, the selection and arrangement of fusion schemes do not have not determined criteria [30]. Further investigations are necessary to design the general framework to combine the fusion algorithm.

(3) Enhancement of the robustness of the model

The proposed method uses many neighboring sources to improve the performance. This method also has limitations. When there are many data sources that are simultaneously interfered, the model may fail. We expect that future research will continue to optimize the model.

## 5. Conclusions

Aiming at the problem of the low accuracy of data credibility assessment, this paper proposed a method for evaluating the credibility of sensor data based on multi-source heterogeneous information fusion with the ARIMA model and the BP neural network. This method considers two factors: the spatio-temporal dependency between data sources and the temporal correlation between time series data. In this paper, the simulation dataset was used for the experiment and the Intel indoor dataset as a benchmark dataset was used to compare the performance. These experiments show that the proposed method, making use of the advantages of multiple sources, has a high evaluation accuracy and a flexible adjustment ability. At the same time, it is also robust to some extent.

## Figures and Tables

**Table 1:** Symbols and descriptions.

Notations	Description
$C_{\mathrm{tr}}^{i}$	Data trend correlation of data sources
$C_{\mathrm{ave}}^{i}$	Data mean aggregation of data source
$S^{i}$	The credibility of the data source
$Tr^{i}$	Data trend function of data source
$F_{}^{i}$	Direct credibility of the data
$M_{}^{i}$	Heterogeneous credibility of data
$D_{}^{i}$	Combined credibility of the data
$Rp_{}^{i}$	Measured value of data source
$Sp_{}^{i}$	Direct prediction data of data source
$Mp_{}^{i}$	Heterogeneous prediction data of data source
$R_{}^{i}(d_{n})$	Comprehensive data credibility of data sources
η	The credibility threshold of the data
Θ	The credibility threshold of the data source

**Table 2:** List of parameters.

Parameters	Description
p	called autoregressive term, also called AR term
q	the number of moving average terms, also called MA terms
d	the difference term, also called the integrated term

**Table 3:** Original data sample.

Time	SD06	SD07	SD09	SD011	THCP04	THCP05	THCP06	SD08
0	0	0	0	0	20	20	20	0
1	0	0	0	0	20.00145	20.00012	20.00012	0
2	0	0	0	0	20.01071	20.00082	20.001	0
3	3.69 × 10^−13^	1.1 × 10^−14^	0	0	20.02051	20.00376	20.00257	0
4	36.85066	0.250725	9.38 × 10^−16^	0	21.35808	20.6181	20.00668	2.49 × 10^−13^
5	80.5879	52.66631	0.000412	4.05 × 10^−13^	28.2894	25.31477	20.01465	27.33613
…	…	…	…	…	…	…	…	…
999	91.59188	93.4295	93.46097	92.45298	49.45999	47.57135	46.13674	94.31777

**Table 4:** Experimental parameter settings.

Parameters	Default Value	Description
$K_{11}$	0.2	Weight coefficient of trend correlation
$K_{12}$	0.8	Weight coefficient of mean aggregation
$K_{21}$	0.4	Weight coefficient of data direct credibility
$K_{22}$	0.6	Weight coefficient of data heterogeneous credibility
η	10	The credibility threshold of the data
Θ	0.5	The credibility threshold of the data source
φ	10	The distance between the target source and other sources.

**Table 5:** Correlation of the signal-node.

Correlation	Temperature	Humidity	Light	Voltage
Temperature	1.00	−0.943	0.687	0.699
Humidity	−0.943	1.00	−0.549	−0.689
Light	0.687	−0.549	1.00	0.41
Voltage	0.699	−0.689	0.41	1.00

**Table 6:** Correlation of the multi-node.

Correlation	Temperature	Humidity	Light	Voltage
No. 13, No. 11	0.905	0.655	0.871	0.912
No. 13, No. 12	0.885	0.850	0.855	0.897
No. 13, No. 14	0.933	0.882	0.902	0.845

**Table 7:** Prediction loss of different models on the two datasets.

Dataset	Model	RSE
Simulation dataset	Heterogeneous network	1.2178
	Homogeneous network	1.7224
Benchmark dataset	MNMF	0.062
	SNMF	0.176
	MSNF	0.141

## Data Availability

Not applicable.
